# Effect of Yuzu (*Citrus junos*) Seed Limonoids and Spermine on Intestinal Microbiota and Hypothalamic Tissue in the Sandhoff Disease Mouse Model

**DOI:** 10.3390/medsci9010017

**Published:** 2021-03-11

**Authors:** Mayumi Minamisawa, Takuma Suzumura, Sudeep Bose, Tetsuyuki Taniai, Gota Kawai, Kyoko Suzuki, Akira Yamaguchi, Shoji Yamanaka

**Affiliations:** 1 Faculty of Advanced Engineering, Chiba Institute of Technology, 2-1-1 Shibazono Narashino, Chiba 275-0023, Japan; drtaniai@mx3.ttcn.ne.jp (T.T.); gota.kawai@p.chibakoudai.jp (G.K.); 2 Graduate School of Engineering, Chiba Institute of Technology, 2-17-1 Tsudanuma Narashino, Chiba 275-0016, Japan; ibcb.takusuzu@gmail.com; 3 Amity Institute of Biotechnology and Molecular Medicine, Amity University, Uttar Pradesh, Noida 201313, India; sbose1@amity.edu; 4 School of Medicine, Yokohama City University, 3-9 Fukuura Kanazawa Kanagawa, Yokohama 236-0004, Japan; ksuzuki@yokohama-cu.ac.jp; 5 Department of Pathology, Yokohama City University, Yokohama 236-0004, Japan; yamaguchiakira@me.com (A.Y.); kowanyamanaka@gmail.com (S.Y.)

**Keywords:** Sandhoff disease, yuzu (*Citrus junos*), limonoids, spermine, intestinal microbiota, next-generation sequencing, short-chain fatty acids, immunoglobulin A

## Abstract

The effect of limonoids and spermine (Spm) extracted from yuzu (*Citrus junos*) seeds on the gut and the brain in a mouse model with Sandhoff disease (SD) was investigated. Wild-type and SD mice were fed a normal diet, or a diet supplemented with limonoid, Spm, or limonoid + Spm for 14–18 weeks, and then 16S rRNA gene amplicon sequencing with extracted DNA from their feces was executed. For SD control mice, intestinal microbiota was mostly composed of *Lactobacillus* and linked to dysbiosis. For SD and wild-type mice fed with limonoids + Spm or limonoids alone, intestinal microbiota was rich in mucin-degrading bacteria, including *Bacteroidetes*, *Verrucomicrobia*, and *Firmicutes*, and displayed a higher production of short-chain fatty acids and immunoglobulin A. Additionally, SD mice fed with limonoids + Spm or limonoids alone had less inflammation in hypothalamic tissues and displayed a greater number of neurons. Administration of limonoids and/or Spm improved the proportions of beneficial intestinal microbiota to host health and reduced neuronal degeneration in SD mice. Yuzu seed limonoids and Spermine may help to maintain the homeostasis of intestinal microbiota and hypothalamic tissue in the SD mouse model.

## 1. Introduction

Lysosomal storage disorders are progressive, single-gene disorders that are characterized by reduced enzyme activity and the accumulation of toxins in cells [1]. Sandhoff disease (SD) is one of a lysosomal storage disorder characterized by the absence of β-hexosaminidase and accumulate of GM2 ganglioside and related glycolipids GA2 in the central nervous system [2,3,4,5]. The glycolipid storage causes severe neurodegeneration through a poorly understood pathogenic mechanism. In SD mice models, extensive oxidative damage has been observed within the caudal regions in the brain [6,7]. No cure for SD has yet been found.

Our team previously demonstrated that treatment with limonoids or spermine (Spm) from yuzu seeds increased survival in SD mice [1]. Recent investigations on the benefits of limonoids or Spm as natural compounds have revealed that they have antitumor, anti-inflammatory, and antioxidant properties [8,9,10], with the Spm notably implicated in maintaining the intestinal mucosal barrier [11]. Although elucidation of the underlying mechanism currently lingers, we consider degeneration of the nervous system to be rooted in oxidative stress and inflammation. Given that diet is well known for moderating these phenomena, consuming limonoids, and spermine (Spm), may be effective at combatting or minimizing neurological damage.

Several studies have shown that bilateral signals between the brain and intestine help to maintain homeostasis within the body and contribute to longevity [12,13,14,15]. Approximately 1000 species and 100 trillion bacteria are present in the human gastrointestinal tract [16], and it is clear that these bacteria play important roles in energy uptake from ingested food [17,18]. The microbiota has been recognized to play a role in human disease, including metabolic [19,20,21,22], inflammatory [23,24,25], and neurodegenerative disorders [26,27,28] and the mechanisms by which these microorganisms contribute to host health [29].

**Figure 1:**
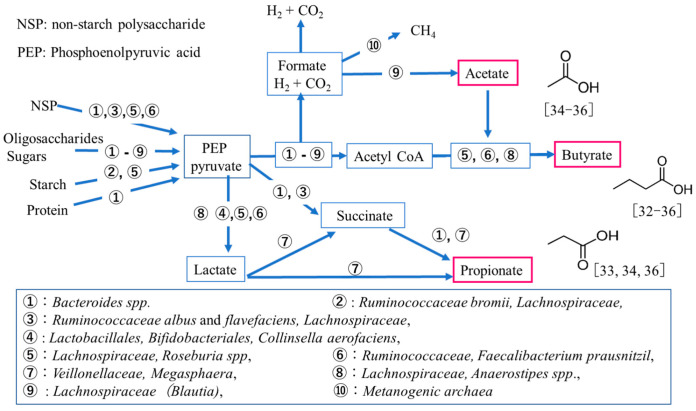
Schematic of intestinal short-chain fatty acid production in human colonic bacteria [30,31,32,33,34,35,36].

Gut microbiome metabolites include short-chain fatty acids (SCFAs), which are a result of intestinal bacterial fermentation of dietary fibers and polysaccharides. A schematic of intestinal SCFA production in humans is shown in Figure 1 [30,31]. The most abundant phyla in the intestine are Bacteroidetes and *Firmicutes*, with members of *Bacteroidetes* mainly producing acetate and propionate and those of *Firmicutes* producing butyrate [32,33]. These intestinal bacterial metabolites have emerged as the quintessential effectors mediating the impact of the microbiome on human or animal physiology [34,35,36]. SCFAs also have been seen to specifically alter hematopoiesis, promote immunoglobulin A(IgA) responses [37], alter T cell homeostasis [38,39], and promote neurogenesis [40]. The potential therapeutic role of SCFAs is also being examined with respect to the gut-brain axis, such as Parkinson’s, autism, and schizophrenia [12,13,14,15]. It is plausible that this gut-brain axis in SD may function by strengthening the production of intestinal microbiota metabolites in SD mice by treating with limonoids and Spm.

Hence, for this investigation, we aimed to examine the composition of intestinal microbiota in SD mice determined by 16S rRNA next-generation sequencing. Correlations of production among microbiota, SCFAs, and IgA were analyzed using SD mice feces. It was investigated the impact of supplementation with limonoids or Spm from yuzu seed extracts on brain lesions, and the lifespan of SD mice.

## 2. Materials and Methods

### 2.1. Materials

The limonoids used in the experiments was extracted and purified from yuzu seed as described in a previous study [41]. Briefly, yuzu seeds were purchased from Kyoto Mizuo (Japan), and a limonoid mixture consisted of deacetylnomilin (105 mg g^−1^ of dry seeds), limonin (95 mg g^−1^ of dry seeds), nomilin (115 mg g^−1^ of dry seeds), and obacnone (17 mg g^−1^ of dry seeds) was extracted and purified.

Determination of the limonoids components and yuzu seed oil was performed by HPLC-MS and GC analysis as described previously [1,41]. Both analyses were conducted using a Shimadzu system (Kyoto, Japan). All standard analytical-grade or extra-pure reagents were purchased from Wako Pure Chemicals Company Ltd. (Osaka, Japan).

### 2.2. SD Mouse Model and Dietary Supplementation with Limonoids and Spm

All mice used in this study were bred and housed under standard non-sterile conditions. All mice including wild-type mice (WT mice) were offspring derived from a pair of Hexa +/−, Hexb +/− mice kindly provided by Richard L. Proia, National Institutes of Health (NIH), Bethesda, MD, USA. Homozygous Hexb knockout mice (Hexb −/−); SD model mice (Hexb −/− mice; C57BL/6×129/Sv background) and WT mice (Hexb +/+ mice) were bred in a closed colony over 30 generations to inbreed for C57BL/6- and 129/Sv-derived genes [20]. SD model mice (*n* = 196: male; *n* = 143, female; *n* = 53) were randomly categorized into four groups (control SD mice; *n* = 101, SD mice fed with limonoids + Spm; *n* = 35, SD mice fed with limonoids alone; *n* = 30, or SD mice fed with Spm alone; *n* = 30). WT mice (*n* = 30, male; 21, female; 9) were randomly categorized into three groups (control WT mice, WT mice fed with limonoids + Spm or limonoids alone; *n* = 10/group, each). A humane endpoint was applied when SD mice became moribund and no longer able to right themselves within 30 s of being laid on their side.

Mice were fed a normal diet or a diet supplemented with limonoid, Spm, or limonoid + Spm from 1 month of age until approximately 14–18 weeks of age when the animals died, according to a method reported previously [1]. Briefly, limonoids (6 mg) were dissolved in yuzu seed oil (1 mL) and sprinkled on mouse chow, and Spm (Sigma-Aldrich, St. Louis, MO, USA) was diluted in drinking water (0.5 mg mL^−1^) to achieve an administration dose of 0.18–0.20 mg g^−1^ of body weight day^−1^. Kaplan–Meier survival analysis for SD mice was performed using the statistical software Easy R (EZR) [42].

### 2.3. Histopathological Analysis of Brain Tissues

Histopathological findings pertaining to the thalamus from control SD mice aged 14–15 weeks were compared with those of treatment SD mice at 16–18 weeks, because of survival extended by limonoids + Spm administration. Formalin-fixed brains were embedded in paraffin, cut into 6 μm sections, and stained with hematoxylin and eosin. The number of neurons was separately counted in the different four fields of the thalamus at 400 × magnification (field size = 0.66 mm^2^). The average of the 4 values obtained in each region was calculated for each mouse, and the total number of neurons remaining in the measured fields is presented as the percentage of the total number of neurons.

### 2.4. Extraction of DNA from Microbiota in Feces

The fecal samples (10–20 mg) were collected until 14–18 weeks of age and suspended in a GTC (4 M guanidium thiocyanate, 100 mM Tris-HCl (pH 9.0), and 40 mM EDTA (pH 8.0)) solution and then homogenized in Lysing Matrix E (MP-Biomedicals, Santa Ana, CA, USA) using a FastPrep FP120 (Thermo Savant, Waltham, MA, USA). Thereafter, DNA (300–360 ng mL^−1^) was extracted from a bead-treated suspension using a phenol-chloroform extraction method and was purified with the FastGeneT Gel/PCR Extraction Kit (NIPPON Genetics Co, Ltd., Tokyo, Japan). All samples were stored at −80 °C until further analysis.

### 2.5. Intestinal Microbiome Analysis: 16S rRNA Amplicon Sequencing Library Preparation

The Illumina protocol 16S Metagenomic Sequencing Library Preparation, Preparing 16S Ribosomal RNA (v4), Gene Amplicons for the Illumina Miseq/MiniSeq System (#15044223JPN Rev. B) was used to construct 16S ribosomal RNA amplicons for the Illumina MiniSeq system (San Diego, CA, USA). The variable V4 regions of 16S rRNA were amplified from bacterial DNA in the feces. PCR was performed using the 16S amplicon PCR forward primer (5′-TCGTCGGCAGCGTCAGATGTGTATAAGAGACAGGTGYCAGCMGCCGCGG TAA-3′) and reverse primer (5′-GTCTCGTGGGCTCGGAGATGTGTATAAGAGACAGGGA CTACHVGGGTWTCTAAT-3′), which were synthesized by Hokkaido System Science Co., Ltd. (Hokkaido, Japan) as the most promising bacterial primer pair. Illumina adapter overhang nucleotide sequences were added at the 5′-end of both primers. Each 25 μL reaction mixture contained 2.5 μL microbial DNA (5 ng μL^−1^), 5 μL of each primer (1 μM), and 12.5 μL of 2× KAPA HiFi HotStart Ready Mix (Kapa Biosystems Ltd., London, UK). A no-template control, in which nuclease-free water was added instead of bacterial DNA, and a negative control, which only contained the extraction from the sample and the stool lysis buffer (ASL buffer), were included in the PCR. The following PCR conditions were used: initial denaturation (95 °C for 3 min), 25 cycles consisting of denaturation (95 °C for 30 s), annealing (55 °C for 30 s), extension (72 °C for 3 min), a final extension step (72 °C for 5 min), and a hold at 4 °C. The resulting PCR products were run on the Agilent 2100 Bioanalyzer (Agilent Technologies, Santa Clara, CA, USA) to verify their sizes, and then they were purified primers and primer dimers using the Agencourt AMPure XP Kit (Beckman Coulter Genomics, Stortford, UK). Dual indices and Illumina sequencing adapters (P5 and P7) were attached to the amplicons using the Nextera XT Index Kit (Illumina) to construct the final libraries. Index PCR was conducted with 50 μL reaction mixtures that contained 5 μL of DNA, 5 μL of Nextera XT Index Primer, 1.5 μL of Nextera XT Index Primer, 2.25 μL of 2× KAPA Hifi HotStart Ready Mix (Kapa Biosystems Ltd.), and 10 μL of nuclease-free water. The following PCR conditions were used: initial denaturation (95 °C for 3 min), eight cycles of denaturation (95 °C for 30 s), annealing (55 °C for 30 s), extension (72 °C for 30 s), a final extension step (72 °C for 5 min), and a hold at 4 °C. Before quantification, the libraries were cleaned using AMPure XP beads (Beckman Coulter Genomics) and amplicon size was verified using the Agilent 2100 Bioanalyzer (Agilent Technologies). All libraries were pooled in equimolar amounts, denatured, and diluted to 1.5 pM before loading onto the MiniSeq flow cell and sequencing on the Illumina MiniSeq platform (Illumina, San Diego, CA, USA).

### 2.6. Microbiota Analysis of Sequencing Data

The raw sequencing files from both amplicon primers were processed using the QIIME 2 pipeline (https://docs.qiime2.org (accessed on 3 July 2019), ver. 2019.7). Preprocessed sequencing reads were used plugins. “Demux” https://github.com/qiime2/q2-demux accessed on 3 July 2019) to import the demultiplexed paired-end sequencing reads and create the “artifact” file (i.e., the qiime2 data format required for subsequent analyses). The “dada2” plugin [43] was applied using the default parameter settings for quality and chimera filtering to trim primers, truncate forward and reverse reads, and collapse reads into representative sequences (i.e., amplicon sequence variants, ASVs). Taxonomy for these ASVs was assigned using the SILVA132 database (https://www.arb-silva.de/documentation/release-132/e_blackstone@me.com (accessed on 13 December 2017)) and “feature-classifier” plugin (<https://github.com/qiime2/q2-feature-classifier> (accessed on 13 December 2017)) with the “fit-classifier-sklearn” method. The taxon summary produced bar plots (<https://github.com/qiime2/q2-taxa> (accessed on 13 December 2017)) according to the sample groupings.

### 2.7. Quantitative Analysis of Short-Chain Fatty Acids (SCFAs) in Feces

The measurement samples of the fecal SCFA content were collected from SD and WT mice at 12–14 weeks of age. The collected feces were stored below −80 °C. Analysis of SCFAs was performed as previously described [23]. Briefly, 100 mg of feces were mixed with 900 mg of 0.5% phosphoric acid solution and then heated at 85 °C for 15 min. After crushing and cooling the sample, the supernatant was obtained by centrifugation at 14,000× *g* for 10 min, mixed with an equal volume of ethyl acetate, and then centrifuged again at 14,000× *g* for 10 min. The ethyl acetate layer was removed, and an internal standard (4-methylvaleric acid) was added. The fecal SCFAs were measured using flame ionization detection (7890 B Gas Chromatograph, Agilent Technologies, Santa Clara, CA, USA) and the internal standard method to determine the proportions of acetic acid, propionic acid, butyric acid, and valeric acid.

### 2.8. Quantitative Analysis of IgA in Feces

The measurement samples of the fecal IgA content were collected from SD and WT mice at 12–14 weeks of age. The collected feces were stored below −80 °C. The measurement sample of the fecal IgA content was prepared by adding PBS with 10 μL of a P8340 protease inhibitor cocktail (Sigma-Aldrich) at a ratio of 1 mL to the mouse feces 0.1 g and then vortexed and centrifuged at 16,000× *g* for 10 min. The fecal IgA content was determined using the E90-103 Mouse IgA ELISA Quantitation Kit (Bethyl Laboratories, Montgomery, TX, USA) and measured on a plate reader (Multiskan FC; Thermo Scientific) at a wavelength of 450 nm.

### 2.9. Statistical Analysis

Kaplan–Meier survival analysis was performed using the statistical software Easy R(EZR) [42]. Data of Kaplan–Meier survival analysis are expressed as mean ± standard error (S.E.). To evaluate significant differences in the relative abundance of bacterial populations, the Kruskal–Wallis test for multiple pairwise comparisons was performed. To conduct comparisons with other experimental parameters, CSV data on bacterial taxonomy was exported from QIIME2 (f taxa-bar-plots.qzv). Continuous parametric data were compared using one-way analysis of variance followed by Tukey’s post-hoc analysis. Unpaired observations were analyzed using a one-sided Student’s *t*-test. The significance threshold was set at *p* < 0.05. Data are expressed as mean ± standard deviation (S.D.). The correlation between the variables of the measured data was analyzed using Pearson’s product-moment correlation coefficient.

## 3. Results

### 3.1. Survival Rates of SD Mice Consuming Limonoids and/or Spm

**Figure 2:**
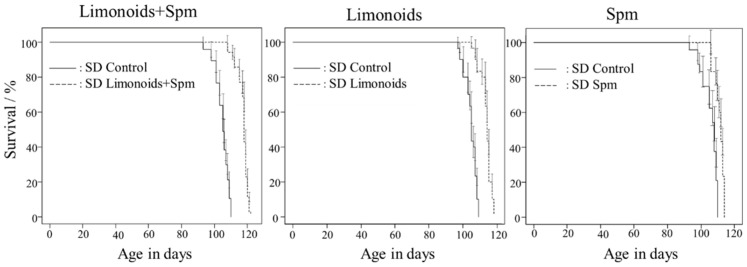
Survival curves for mice with Sandhoff disease fed with limonoids + Spm, limonoids alone, or Spm alone. Compared with the control group, survival rates were significantly different for all treatment groups: control (*p* < 0.001; *n* = 101), limonoids + Spm (*p* < 0.001; *n* = 35), limonoids (*p* < 0.001; *n* = 30), or Spm (*p* < 0.001; *n* = 30). Data are expressed as mean ± S.E. of the mean.

SD control mice had a mean life span of 105 ± 0.25 (mean ± S.E.) days. Figure 2 shows that treatment with either limonoids or Spm starting at 4 weeks of age extended survival to 114 ± 0.61 or 111 ± 0.50 (mean ± S.E.) days, respectively, and resulted in a 5% to 10% increase in life span (*p* < 0.001). Treatment with limonoids + Spm starting at 4 weeks of age extended survival to 118 ± 0.57 (mean ± S.E.) days and resulted in a 12% increase in life span (*p* < 0.001). Compared with the SD control mice, the survival rates for all treatment groups were significantly different (*p* < 0.001).

### 3.2. Histopathological Analysis of Brain Tissue

**Figure 3:**
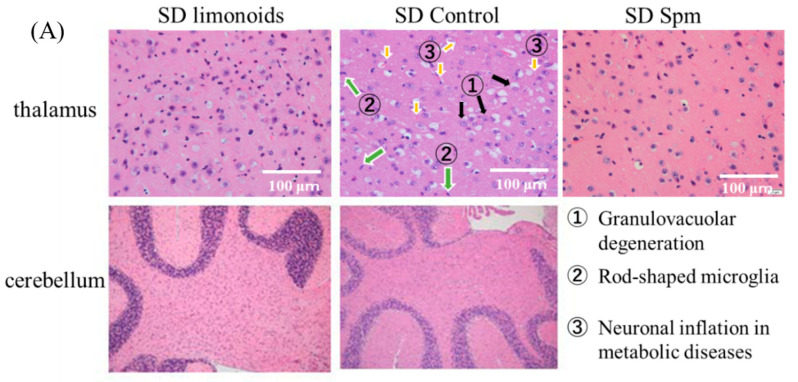

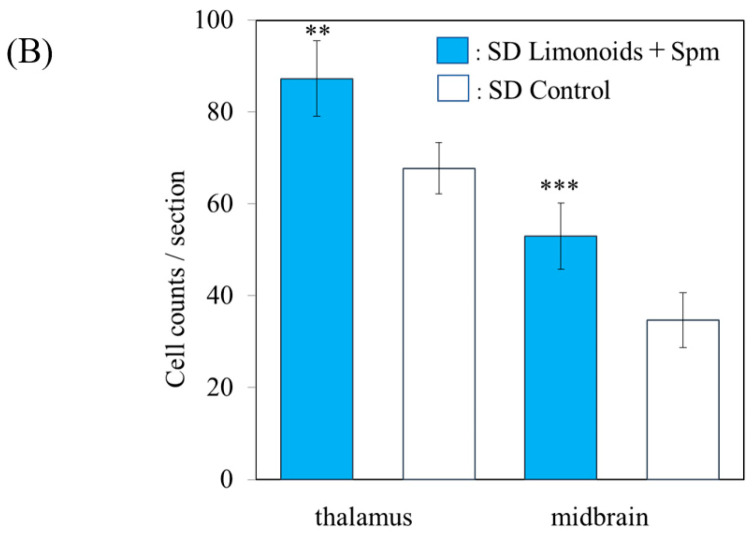
Histopathology of brain tissues for mice with Sandhoff disease (SD) fed with limonoids and Spm. (**A**) H&E staining of thalamic (original magnification: ×400, field size; 0.66 mm^2^) and cerebellum sections obtained from SD mice fed with limonoids, Spm or a normal diet (SD controls). The sections show enlarged cells with ganglioside storage. (**B**) Number of neurons in the thalamus and midbrain for SD mice fed with limonoids + Spm or control SD mice. Values represent means ± S.D. (*n* = 4). Data onto a bar graph are plotted in comparison to the controls for clarity; ** *p* ≤ 0.01; *** *p* ≤ 0.001.

Figure 3A shows that histopathological examination of thalamic samples from SD mice demonstrated typical gangliosidosis and inflammatory/autoimmune disease (https://rarediseases.info.nih.gov/diseases/7604/sandhoff-disease (accessed on 7 March 2021)) as characterized by fat accumulation, granulovacuolar degeneration, rod-shaped microglia, and neuronal inflammation, as outlined by the Tokyo Metropolitan Institute for Medical Science (https://igakuken.or.jp/ (accessed on 7 March 2021)). Mice fed with limonoids + Spm not only exhibited a decrease in these characteristic histopathological degenerations that was consistent with previous studies on mice fed with limonoids or Spm [1], but they also experienced minimal degeneration of the vacuoles, Purkinje cells, and the granular layer of the cerebellum. The number of neurons in the thalamus and midbrains in SD mice fed with limonoid and Spm were 1.3-fold (*p* < 0.01) and 1.5-fold (*p* < 0.001) higher, respectively, than those in SD control mice (Figure 3b).

### 3.3. Composition of Fecal Microbiota in SD and Wild Control (WT) Mice

**Figure 4:**
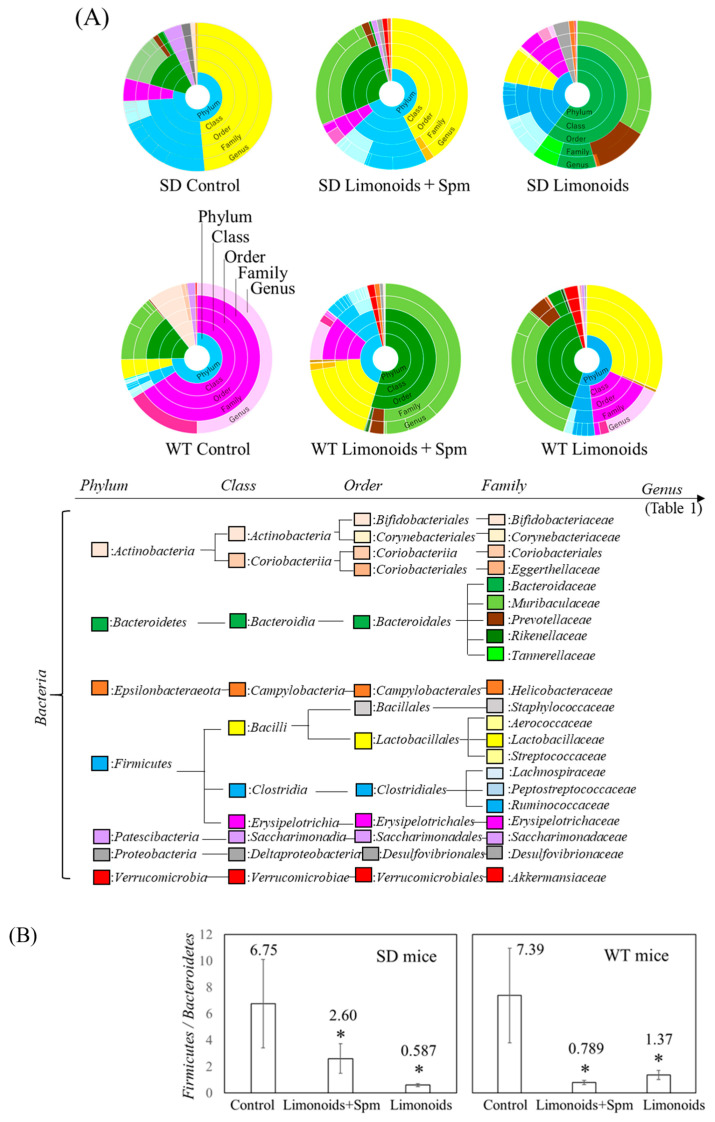
Estimated ratios (%) of phylum–genus taxonomic categories identified by genomic analysis of 16S rRNA in feces of mice aged 12–14 weeks (**A**), and relative abundance *Firmicutes/Bacteroidetes* ratio (**B**). Values represent mean ± S.D. SD and WT mice were treated at 12–14 weeks of age (WT mice; *n* = 9/each group, SD mice; *n* = 15/each group). Data are plotted in comparison to the controls for clarity; * *p* ≤ 0.05. SD, Sandhoff disease; WT, wild-type.

Figure 4A shows that the phylum to genus taxonomic classifications, as determined by 16S rRNA gene amplicon sequencing, was quite different for the SD and WT mice. In the feces *Bacteroidetes* and *Firmicutes* accounted for most of the phylum, and the abundances of *Firmicutes* and *Bacteroidales* were smaller and larger, respectively, in feces for SD and WT mice fed with limonoids + Spm and limonoids alone. Figure 4B shows that the *Firmicutes/Bacteroidetes* ratio in the SD and WT control mice was high and nearly the same (SD, 6.75 ± 3.34; WT, 7.39 ± 3.57). This ratio was significantly reduced in SD and WT mice fed with limonoids + Spm (SD, 2.60 ± 1.11; WT, 0.789 ± 0.159; *p* < 0.05, compared with the respective control group) or those fed with limonoids alone (SD, 0.587 ± 0.107; WT, 1.37 ± 0.159; *p* < 0.05).

**Figure 5:**
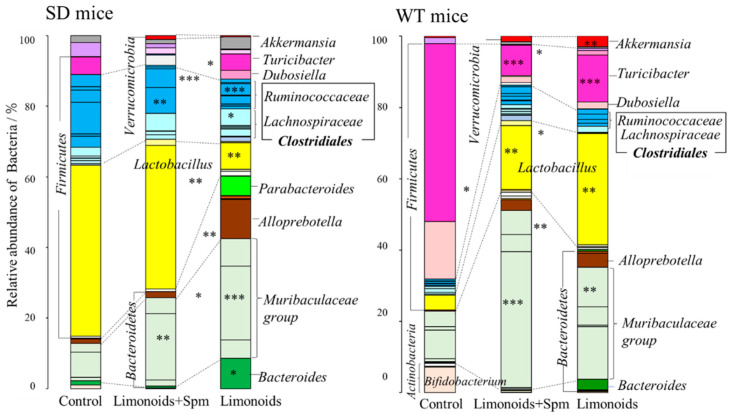
Estimated ratios (%) of taxonomic categories at the genus level were identified according to genomic analysis of 16S rRNA in the feces of SD or WT mice aged 12–14 weeks (WT mice; *n* = 9/each group, SD mice; *n* = 15/each group). Values represent means ± S.D. Data onto a bar graph are plotted in comparison to the controls for clarity; * *p* ≤ 0.05; ** *p* ≤ 0.01; *** *p* ≤ 0.001. SD, Sandhoff disease; WT, wild-type.

Figure 5 and Table 1 present a comparison of the relative abundance of bacterial (%) at the genus level between SD and WT control mice and mice fed with limonoids + Spm. The abundance of *Lactobacillus* was similar in the SD control mice and SD mice fed with limonoids + Spm (47% ± 11% vs. 39% ± 12%); SD mice fed with limonoids alone displayed a lower abundance of *Lactobacillus* (7.4% ± 1.8%). Moreover, the abundance of microbiota classified in the phylum *Firmicutes*, order *Clostridiales*, and genus *Ruminococcaceae* significantly decreased in SD mice fed with limonoids + Spm or limonoids compared with SD control mice. By contrast, the abundance of microbiota classified in the phylum *Firmicutes*, order *Clostridiales*, and genus *Lachnospiraceae*, as well as that of microbiota classified in the phylum *Bacteroidetes*, order *Bacteroidales*, and genera *Muribaculaceae*, *Parabacteroroides*, *Alloprebotella*, and *Bacteroides*, was significantly increased in the SD mice fed with limonoids + Spm or limonoids.

Although the *Firmicutes*/*Bacteroidetes* ratio was similar at the phylum level, WT mice had different microbiota composition than SD mice at the genus level. In WT control mice, the abundances of *Turicibacter* (50% ± 6.3%) and *Dubosiella* (23.29% ± 4.90%) in the *Erysipelotrichaceae* family (*Firmicutes*) and *Bifidobacterium* (7.2% ± 0.026%) in the *Actinobacteria* phylum significantly increased. *Dubosiella* was not listed in the 2017 genomic database, which was a recently proposed novel genus [44]. The members of *Erysipelotrichaceae* family occupied the majority the abundance of microbiota in feces from WT control mice. It is reported that the abundances of members of *Erysipelotrichaceae* family increased due to fat accumulation from normal diet in mice [1,42].

In contrast to SD mice, WT mice showed an increased abundance of *Lactobacillus* in the following order: control (4.2% ± 1.5%) < limonoids + Spm (18% ± 5.7%) < limonoids alone (31% ± 9.8%). In both SD and WT mice, the *Verrucomicrobia* phylum (*Akkermansia* genus) was observed in microbiota mainly from mice fed with limonoids + Spm or limonoids alone. An intriguing observation was that there was a higher abundance of mucin-degrading bacteria in mice fed with limonoids alone. Moreover, various bacteria involved in mucin metabolism for intestinal epithelial cells were present, including *Bacteroides*, *Ruminococcus*, *Prevotella*, and *Desulfofibrio*.

### 3.4. SCFAs and IgA Productions in SD and WT Mice Feces

**Figure 6:**
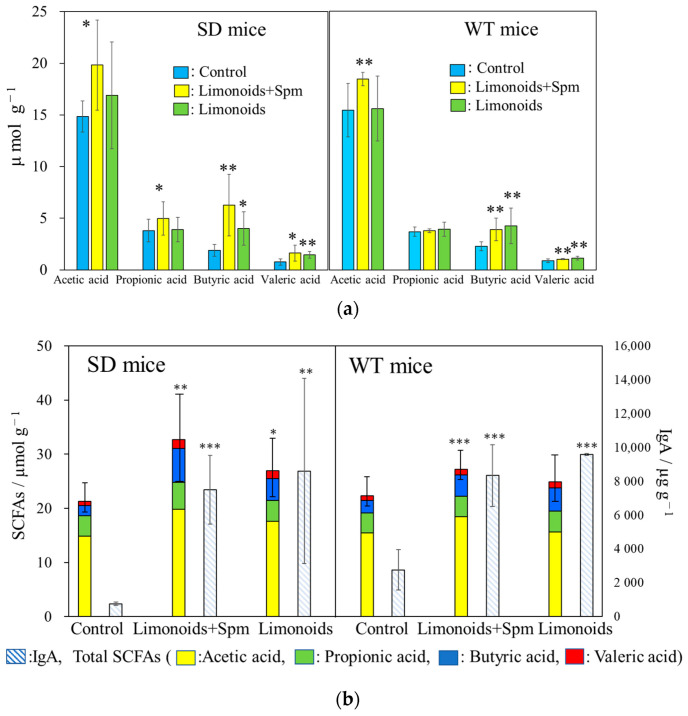
Short-chain fatty acid (SCFA) content (µmol g^−1^) (**a**) in feces from SD or WT mice (**b**) and comparison of immunoglobulin A (IgA) production (µg g^−1^) vs. total SCFA content. SD and WT mice were treated at 12–14 weeks of age (WT mice; *n* = 9/each group, SD mice; *n* = 15/each group). Values represent means ± S.D. Data onto a bar graph are plotted in comparison to the controls for clarity; * *p* ≤ 0.05; ** *p* ≤ 0.01; *** *p* ≤ 0.001. The correlation coefficient between the levels of SCFAs and IgA, *r* = 0.52, *p* 0.05. SD, Sandhoff disease; WT, wild-type.

Figure 6a shows SCFAs content (µmol g^−1^) in feces from SD and WT mice at 12–14 weeks of age. Production of acetic acid was highest among SD and WT mice fed with limonoids + Spm, production of butyric acid was highest among SD and WT mice fed with limonoids + Spm or limonoids alone, and production of propionic acid was similar across all groups. The reduction in SCFA content was the highest in SD and WT control mice.

Figure 6b show the comparison of immunoglobulin A (IgA) production (µg g^−1^) vs. total SCFA content in feces from SD or WT mice at 12–14 weeks of age. IgA production was significantly higher in mice fed with limonoids + Spm (SD, 7700 ± 1241 μg g^−1^, *p* < 0.001; WT, 8492 ± 1096 μg g^−1^, *p* < 0.001) or limonoids alone (SD, 8596 ± 3160 μg g^−1^, *p* < 0.01; WT, 95611 ± 48 μg g^−1^, *p* < 0.001) than in control mice (SD, 753 ± 102 μg g^−1^; WT, 2066 ± 996 μg g^−1^). The amounts of IgA production increased by fourfold to tenfold in mice fed with limonoids + Spm or limonoids alone than SD and WT control mice. A positive correlation was observed between the production levels of SCFAs and IgA, *r* = 0.52, *p* < 0.01.

### 3.5. Correlation between Limonoids and/or Spermine and the Clostridiales Group

**Figure 7:**
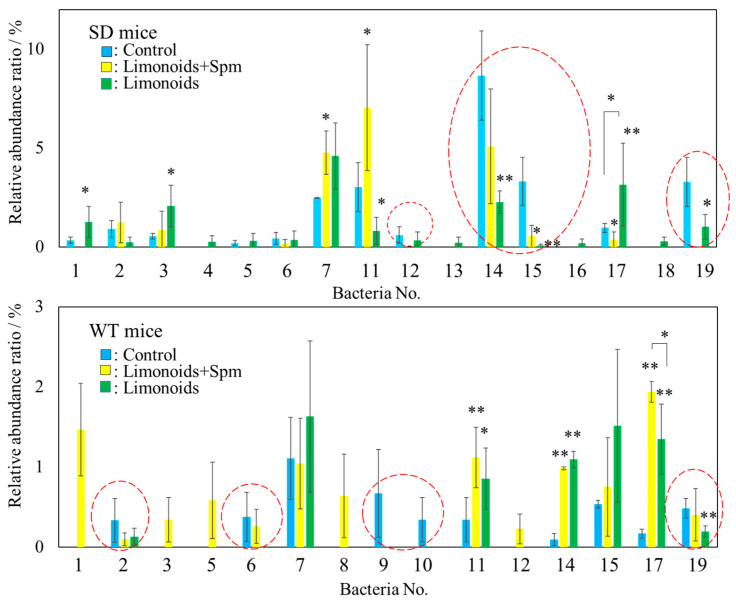
Comparison of the relative abundance (%) of bacteria at the genus level belonging to the *Clostridiales* order in feces for SD or WT mice. The bacterial numbers in the figure correspond to the bacterial numbers listed in Table 1. Bacteria whose relative abundance ratio was suppressed by feeding limonoids + Spm or limonoids alone are surrounded by a red dashed line. Values represent mean ± S.D. Data are plotted in comparison to the controls for clarity; * *p* ≤ 0.05; ** *p* ≤ 0.01. SD, Sandhoff disease; WT, wild-type.

**Figure 8:**
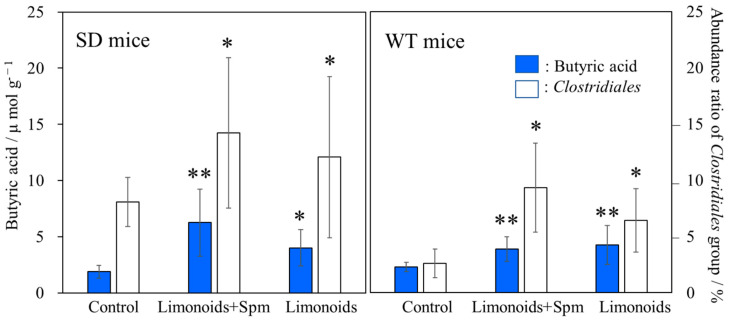
Comparison of the amount of butyric acid in feces from SD or WT mice (μmol g^−1^) and relative abundance (%) of bacteria (*Clostridiales* groups) identified from Figure 7 as promoting butyric acid metabolism. Values represent mean ± S.D. Data onto a bar graph are plotted in comparison to the controls for clarity; * *p* ≤ 0.05; ** *p* ≤ 0.01. The correlation coefficient between the relative abundance (%) of the *Clostridiales* groups and the amount of butyric acid, *r* = 0.72, *p* < 0.01. SD, Sandhoff disease; WT, wild-type.

We found a significant difference SCFAs content in mice feces between SD or WT control mice and those that fed limonoids + Spm; in particular, butyric acid production increased in SD mice fed limonoids + Spm (Figure 6a). To identify intestinal microbiota that might be involved in butyric acid metabolism, we focused on bacteria belonging to the *Clostridiales* group. A significantly large relative abundance ratio of bacteria in the *Clostridiales* group was found in mice feces fed with limonoids + Spm or limonoids alone (Figure 7, red dashed line; Table 1, and Figure 8).

We found that in SD mice fed with limonoids + Spm or limonoids alone, the abundance of *Ruminiclostridium 9* (No. 12), *Ruminococcaceae UCG 14* (No. 14), *Ruminococcus 1* (No. 15), and *Ruminococcaceae* (No. 19) was significantly altered across the three groups of SD mice. Furthermore, SD mice fed with limonoids + Spm showed a significant increase in the levels of *Ruminiclostridium 5* (No. 11) compared with SD control mice or those fed with limonoids alone. By contrast, the number of bacterial types of SD mice fed with limonoids alone increased 136% (*p* < 0.001) vs. control or 120% (*p* < 0.01) vs. limnoids + Spm. The members of the *Clostridiales* order were significantly different between WT control mice and mice fed with limonoids + Spm or limonoids alone, though nearly the same bacterial populations were identified in SD and WT mice fed with limonoids + Spm or limonoids alone. However, the abundance ratio of bacteria identified differed. The relative abundance ratio of *Clostridiales* in feces of SD or WT mice fed with limonoids + Spm increased 135% (*p* < 0.001) vs. SD mice fed limonoids or 134% (*p* < 0.05) vs. WT fed limonoids.

From Figure 7 and Table 1, the relative abundance ratio (%) of the *Clostridiales* group bacteria identified as promoting butyric acid metabolism in the presence of limonoids + Spm or limonoids alone was compared with the amount of butyric acid measured in mouse feces (μmol g^−1^) (Figure 8). A positive correlation was shown between the abundance of bacteria belonging to the *Clostridiales* order and butyric acid production promoted by the consumption of limonoids + Spm or limonoids alone (r = 0.72, *p* < 0.01). We found that the relative abundance ratio of the *Clostridiales* order group found in the feces of both SD and WT mice fed limonoids + Spm were larger than other mice.

## 4. Discussion

In the current study, we found that SD mice fed with limonoids, Spm, or limonoids + Spm extracted from yuzu seeds displayed a slower rate of disease progression compared with SD control mice. Noteworthily, the brains of SD mice fed with limonoids and Spm had less degeneration with inflammation, suggesting that limonoids and Spm suppress inflammation caused by glycolipid accumulation in the central nervous system.

The *Firmicutes/Bacteroidetes* ratio was similar in SD and WT control mice compared with the other groups; however, the microbiota at the genus level for SD control mice was composed of 47% *Lactobacillus*, whereas the WT control mice existed 50% *Turicibacter* and 16% *Dubosiella* belonging *Erysipelotrichaceae* family. It is reported that the increase in abundances of members of *Erysipelotrichaceae* family is due to fat accumulation from the normal diet in mice [1,42].

While there is generally known the mechanisms via which gut microbiota may mediate obesity, the identities of the bacteria that constitute an “obese microbiota” such as *Erysipelotrichaceae* have not yet been unequivocally established. The feature is that the *Firmicutes*/*Bacteroidetes* ratio increases as the obese microbiota increases [42,43]. This phenomenon was similarly confirmed in WT control mice. Actually, in SD control mice, *Turicibacter* existed only 4.8%.

On the other hand, such extremely biased ratios of *Lactobacillus* in SD control mice may be implicated in dysbiosis, a condition in which microbiota diversity is reduced primarily because of antibiotic administration, unhealthy dietary intakes, infection, genetic abnormalities, and immune disorders [45]. Dysbiosis has also been reported to increase the permeability of epithelial cells in the small intestine [46], and ulcerations attributed to increased permeability of these cells can lead to intestinal inflammation via intestinal microbiota or their associated metabolites. Examples of such dysbiosis have been reported in the case of Parkinson’s disease. In an analysis of intestinal microbiota from patients with Parkinson’s disease and mouse model, amounts of *Clostridium* and *Bacteroides* were significantly lower and amounts of *Lactobacillus* were significantly higher compared with healthy counterparts, the ratios of these bacterial populations are reported as the prime indicants of dysbiosis in Parkinson’s disease [47,48,49,50]. Furthermore, untreated patients with Parkinson’s disease have increased colonic permeability, and that patients have a pathological expression of α-synuclein in their colon. Even may initiate Parkinson’s disease events through gut-derived, lipopolysaccharide-induced neuronal injury. It has been suggested that the intestinal plexus becomes abnormal and ultimately affects the central nervous system [47,48,49,50,51]. Although the mechanisms underlying increased intestinal permeability in dysbiosis remain elusive [52], such increased permeability will likely render intestinal neuronal cells sensitive to the intestinal microbiota.

In SD mice, supplementing the diet with limonoids + Spm or limonoids alone induced many changes in the relative abundance of bacterial species in the intestinal microbiota for SD mice. Importantly, these compounds alleviated intestinal dysbiosis by promoting the growth of *Lachnospiraceae*, *Alloprevotella*, *Akkermansia*, and *Bacteroides*, as well as suppressing the growth of *Lactobacillus*. While, in WT mice, supplementing the diet with limonoids + Spm or limonoids alone increased the diversity of the gut microbiome by suppressing the growth of *Turicibacter* and *Dubossiella*. Recently, *Akkermansia* has attracted attention as a genus that suppresses inflammation, improves insulin resistance [53], and modulates susceptibility to seizure in refractory epilepsy [54].

*Akkermansia, Bacteroides,* and *Bifidobacterium* are called mucolytic bacteria, because, they have dozens of glycosidases and peptidases, decompose mucin glycoproteins, and can use as their own nutrient source. Mucolytic bacteria (mucin-degrading bacterium) are a general term for bacteria that decompose gastrointestinal tract mucin and use it as a carbon source. Mucolytic bacteria are present in healthy humans and animals. Glycoproteins are major players in the mucus protective barrier in the gastrointestinal and other mucosal surfaces. In particular, the mucins, are responsible for the protective gel barrier. Mucus layers provide a physical barrier of protection from the microbiota in the large intestine, though there needs to be a functional interplay between this layer and mucin-degrading or mucin-utilizing bacteria in the intestinal microbiota for proper homeostasis [55]. Sialic acid produced by mucin decomposition is used as a carbon source for specific bacteria, and in the intestine, the sialic acid supplier and the user bacterium coexist in an exquisite balance [56]. It has also been suggested that the cross-feeding of degradation products “strategies through the expansion of carbohydrate acquisition abilities by gut bacteria” occurs between symbiotic bacteria [55,56,57].

On the other hand, if this symbiotic relationship is disrupted and the amount of free sialic acid in the intestine changes, the risk of infection by pathogenic bacteria increases [56]. In other words, the presence of a rich mucin layer is essential for maintaining diversity in the intestinal microbiota [55,56,57]. In this study, diet supplementation with limonoids + Spm or limonoids alone yielded an increase in the number of mucin-degrading bacteria. However, SD mice fed with limonoids + Spm survived longer than SD control mice, SD mice fed with limonoids or Spm alone. Especially, the life-extension rate (%) of mice fed with Spm alone was less than 50% of that for mice fed with limonoid + Spm. In view of the intestinal microbiota profiles of the groups, these results suggest that the effects of Spm and limonoids on survival rates and/or the intestinal microbiota in this model are different, which is why we pursued the composition of enterobacteria and their metabolites in greater detail.

**Figure 9:**
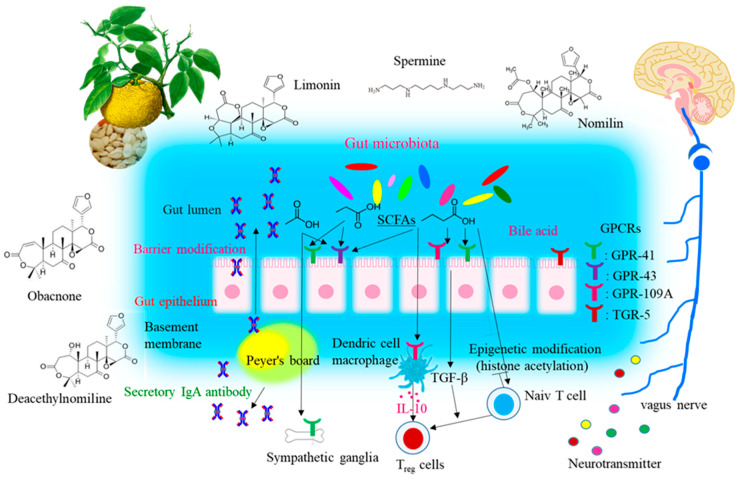
Conceptual diagram of immune responses of intestinal microbiota in the colon of SD and WT mice fed with limonoids + spermine or limonoids alone [56,57,58,59,60,61,62,63,64,65,66].

SCFAs, one of the enterobacteria metabolites, are known to significantly affect host immunity [58]. The immune response induced by SCFAs and their receptors may be one of the primary strategies to maintain the health of the host (Figure 9). SCFAs are sensed by host cells through various G-protein coupled receptors (GPRs), such as GPR41, GPR43, and GPR109A, known as free fatty acid receptors, and the intracellular receptor [59]. GPR41 and GPR43, which suppress appetite and host insulin sensitivity [60], are SCFA receptors that have been recently identified in enteroendocrine cells [61,62,63] and correspond primarily to levels of acetic acid, propionic acid, and butyric acid. Acetic acid induces the activation of gastrointestinal inflammasomes through GPR43 and GPR109A [64,65], and it has also been reported that propionic acid causes proliferation of dendritic precursors in the bone marrow through GPR41, as well as acts to express molecules necessary for B cell differentiation to alter IgA production [66]. We showed that the addition of Spm to the diet clearly increased the proportion of *Clostridiales* and butyric acid in feces.

Butyric acid exhibits remarkable differentiation-inducing activity to regulatory T cells [22]. T cell induction, potentially through a mechanism that involves the production of SCFAs, acts through both immune and nonimmune cells to induce Foxp3 expression in CD4 T cells [21,22]. Foxp3 T cells comprise a large fraction of CD4 T cells in the small and large intestines and have critical functions in the maintenance of tolerance toward commensal microbiota and food antigens [67,68]. Although a fraction of intestinal T cells are derived from the thymus, many others are induced from SCFAs metabolized by commensal microbiota.

IgA helps colonization indigenous bacteria, although eliminated by infectious bacteria. We found that when SD and WT mice are fed with limonoids, the relative abundance ratio of mucolytic bacteria species such as *Akkermansia*, *Bacteroides*, and *Bifidobacterium* increased in these mice feses.

Some bacteria, like *Bacteroides*, have shown to have anti-inflammatory properties that are beneficial to the host. IgA secreted into the intestinal tract not only functions as an antibody responsible for eliminating pathogens and neutralizing toxins but also binds to *Bacteroides* through a polysaccharide and other bacterial species belonging to *Firmicutes* in the intestinal mucosa [69,70,71]. *Bacteroides* can coat themselves with IgA and become established in the intestinal mucus layer of the host. It is suggested that these interact and change the composition and metabolic function of the collective microbiota [69]. SCFAs absorbed from the large intestine and transferred to the blood also act on the immune cells of Peyer’s patches in the small intestine, enhancing IgA production in the small intestine [70]. It is estimated that the small intestine produces several times the amount of IgA produced in the large intestine, but it is a prerequisite that a sufficient amount of SCFAs is produced by the metabolic activity of the intestinal microbiota [71,72]. In our investigation, it was suggested that SCFAs and IgA productions were significantly higher in mice fed with limonoids + Spm or limonoids alone than in SD and WT control mice. A positive correlation also was shown between the production levels of SCFAs and IgA.

Recently, it was reported that some plasma cells (PC) in the CNS of mice with experimental autoimmune encephalomyelitis (EAE) originate in the gut and produce IgA. They showed that IgA + PC are dramatically reduced in the gut during EAE, and a reduction in IgA-bound fecal bacteria is seen in MS patients during disease relapse. Moreover, it was reported removal of plasmablast (PB) plus PC resulted in exacerbated EAE that was normalized by the introduction of gut-derived IgA + PC. They are suggested that IgA + PB and/or PC mobilized from the gut may be playing in suppressing neuroinflammation [73].

Therefore, it suggests that when SD mice are fed with limonoids + Spm or limonoids, the mucus layer in the mouse Intestines was maintaining as a carbon and energy source to the intestinal microbiota [56]. As a result, it can be considered that the abundant SCFAs produced via the metabolism of the gut microbiome were abundantly absorbed by the host to more enhance the barrier function, enhancing IgA production. Recently, it was reported that some plasma cells (PC) in the CNS of mice with experimental autoimmune encephalomyelitis (EAE) originate in the gut and produce IgA. They showed that IgA + PC are dramatically reduced in the gut during EAE, and a reduction in IgA-bound fecal bacteria is seen in MS patients during disease relapse. Moreover, it was reported removal of plasmablast (PB) plus PC resulted in exacerbated EAE that was normalized by the introduction of gut-derived IgA + PC. They are suggested that IgA + PB and/or PC mobilized from the gut may be playing in suppressing neuroinflammation [73].

Most importantly, it was observed at the same time by the combination diet of limonoids + Spm the maintenance of intestinal homeostasis and the suppression of inflammation in the brain of SD mice. Thus, our study that SCFAs and IgA production promoted by feeding limonoids + Spm in SD mice showed a correlation, suggests that the above immune response may be induced.

## 5. Conclusions

In this study, it has been observed neurodegeneration in the brains and dysbiosis of the intestine microbiome for SD control mice. Brain neurodegeneration and the diversity of intestinal microbiota involved in host intestinal homeostasis in SD mice were improved by ingesting the combination diet of limonoids and Spermine. These results suggest that yuzu seed limonoids and Spermine may help to maintain the homeostasis of intestinal microbiota and hypothalamic tissue in the sandhoff disease mouse model.

## Figures and Tables

**Table 1:** Comparison of the relative abundance of bacterial (%) in feces from SD or WT mice fed with a normal diet (control), limonoids + Spm or limonoids alone.

Genus Classification	Relative Abundance of Bacterial (%)					
	SD Mice			WT Mice		
	Control	Limonoids + Spm	Limonoids	Control	Limonoids + Spm	Limonoids
No. *Firmicutes*–*Clostridiales*–*Lachnospiraceae* family						
1. *Lachnoclostridium*	0.33 ± 0.15	nd.	1.3 ± 0.79 *	nd.	1.5 ± 0.58	nd.
2. *Lachnospiraceae FCS020 group*	0.91 ± 0.42	1.2 ± 1.0	0.22 ± 0.27	0.34 ± 0.27	0.10 ± 0.08	0.13 ± 0.10
3. *Lachnospiraceae NK4A136 group*	0.54 ± 0.15	0.84 ± 0.96	2.1 ± 1.1 *	nd.	0.34 ± 0.20	nd.
4. *Lachnospiraceae UCG-004*	nd.	nd.	0.26 ± 0.31	nd.	nd.	nd.
5. *Lachnospiraceae UCG-006*	0.19 ± 0.14	nd.	0.31 ± 0.38	nd.	0.58 ± 0.48	nd.
6. Uncultured (*Lachnospiraceae*)	0.43 ± 0.31	0.16 ± 0.22	0.36 ± 0.44	0.37 ± 0.30	0.26 ± 0.21	nd.
7. *Lachnospiraceae*	2.5 ± 0.018	4.8 ± 1.1 *	4.6 ± 1.7 *	1.1 ± 0.51	1.0 ± 0.56	1.6 ± 0.94
8. *Eubacterium fissicatena group*	nd.	nd.	nd.	nd.	0.64 ± 0.52	nd.
*Firmicutes*–*Clostridiales*–*Peptostreptococcaceae* family						
9. *Romboutsia*	nd.	nd.	nd.	0.67 ± 0.55	nd.	nd.
10. *Peptostrepto- coccaceae*	nd.	nd.	nd.	0.34 ± 0.28	nd.	nd.
*Firmicutes*–*Clostridiales*–*Ruminococcaceae* family						
11. *Ruminiclostridia- um 5*	3.0 ± 1.2	7.0 ± 3.2 **	0.81 ± 0.68	0.34 ± 0.28	1.1 ± 0.34	0.85 ± 0.38
12. *Rumini- clostridium 9*	0.60 ± 0.42	0.34 ± 0.41	nd.	nd.	0.23 ± 0.19	nd.
13. *Ruminococcaceae* *UCG-009*	nd.	nd.	0.22 ± 0.27	nd.	nd.	nd.
14. *Ruminococcaceae UCG-014*	8.7 ± 2.2	5.1 ± 2.9	2.3 ± 0.58	0.09 ± 0.08	0.98 ± 0.02	1.1 ± 0.10
15. *Ruminococcus 1*	3.3 ± 1.2	0.54 ± 0.56	0.062 ± 0.07	0.53 ± 0.05	0.75 ± 0.61	1.5 ± 0.95
16. *UBA1819* (*Ruminococcaceae*)	nd.	nd.	0.18 ± 0.22	nd.	nd.	nd.
17. *Eubacteriumcopro-stanoligenes group*	0.96 ± 0.23	0.35 ± 0.39	3.1 ± 2.1	0.17 ± 0.05	1.9 ± 0.13	1.3 ± 0.43
18. Uncultured (*Ruminococcaceae*)	nd.	nd.	0.29 ± 0.20	nd.	nd.	0.29 ± 0.20
19. *Ruminococcaceae*	3.3 ± 1.2	nd.	1.0 ± 0.62	0.49 ± 0.12	0.40 ± 0.33	0.19 ± 0.07
*Firmicutes*–*Lactobacillales*–*Lactobacillaceae* family						
*Lactobacillus*	47 ± 11	39 ± 12	7.4 ± 1.8 **	4.2 ± 1.5	18 ± 5.7 **	31 ± 9.8 **
*Lactococcus*	nd.	1.7 ± 2.0	0.30 ± 0.37	nd.	1.5 ± 1.2	0.19 ± 0.16
*Firmicutes*–*Erysipelotrichales*–*Erysipelotrichaceae* family						
*Candidatus Stoquefichus*	nd.	2.9 ± 3.4	nd.	nd.	0.78 ± 0.33	nd.
*Dubosiella*	nd.	nd.	2.4 ± 1.4	16 ± 1.9	1.7 ± 1.4 ***	1.9 ± 1.5 ***
*Erysipelatoclostridium*	nd.	0.28 ± 0.39	nd.	nd.	0.29 ± 0.23	nd.
*Turicibacter*	4.8 ± 1.9	nd.	4.5 ± 2.1	50 ± 6.3	8.5 ± 4.0 ***	13 ± 6.2 ***
*Erysipelotrichaceae*	0.06 ± 0.04	1.7 ± 2.4	1.2 ± 0.92	nd.	0.23 ± 0.18	1.3 ± 0.55
*Patescibacteria*–*Saccharimonadales*–*Saccharimonadaceae* family						
*Candidatus Saccharimonas*	3.8 ± 2.7	1.2 ± 1.6	0.22 ± 0.27	1.8 ± 1.4	nd.	0.68 ± 0.16
*Proteobacteria–Desulfovibrionales–Desulfovibrionaceae* family						
*Desulfovibrio*	1.9 ± 0.53	1.1 ± 1.5	3.4 ± 3.9	nd.	0.75 ± 0.60	0.38 ± 0.03
*Verrucomicrobia*–*Verrucomicrobiales*–*Akkermansiaceae* family						
*^a^ Akkermansia*	nd.	1.1 ± 1.3	0.31 ± 0.27	0.37 ± 0.30	1.6 ± 1.3 *	3.0 ± 2.0 ***
*Bacteroidetes*–*Bacteroidales*–*Bacteroidaceae* family						
*^a^ Bacteroides*	1.2 ± 0.076	0.51 ± 0.36	8.4 ± 4.5 *	0.14 ± 0.11	0.21 ± 0.13	3.0 ± 0.02
*Bacteroidetes*–*Bacteroidales*–*Muribaculaceae* family						
*Muribaculum*	0.90 ± 0.64	1.6 ± 2.3 **	5.1 ± 5.8 ***	0.90 ± 0.73	0.35 ± 0.26	15 ± 3.4 ***
Uncultured *Bacteroidales bacterium*	nd.	nd.	nd.	nd.	0.26 ± 0.09	nd.
Uncultured *Barnesiella sp*.	nd.	nd.	nd.	nd.	nd.	0.41 ± 0.34
Uncultured *Bacterium*	6.9 ± 2.7	26 ± 3.8 **	28 ± 9.5 ***	8.0 ± 5.5	38 ± 2.9 ***	5.1 ± 0.53
Uncultured *organism*	nd.	nd.	nd.	1.0 ± 0.82	4.8 ± 0.76	nd.
*Muribaculaceae*	2.4 ± 0.097	6.6 ± 0.27	7.5 ± 2.3	4.4 ± 1.6	6.8 ± 0.22	11 ± 2.7 **
*Bacteroidetes*–*Bacteroidales*–*Prevotellaceae* family						
*^a^ Alloprevotella*	1.2 ± 0.37	1.6 ± 0.63	11 ± 2.7	0.30 ± 0.24	3.0 ± 0.56	4.0 ± 1.6
*^a^ Paraprevotella*	nd.	nd.	0.18 ± 0.11	nd.	0.10 ± 0.08	0.53 ± 0.34
*^a^ Prevotellaceae NK3B31 group*	nd.	nd.	0.089 ± 0.11	nd.	0.17 ± 0.14	nd.
*^a^ Prevotellaceae*	nd.	nd.	0.65 ± 0.80	nd.	nd.	0.28 ± 0.23
*Bacteroidetes*–*Bacteroidales*–*Tannerellaceae* family						
*^a^ Parabacteroides*	nd.	nd.	5.4 ± 1.5	nd.	0.73 ± 0.57	0.58 ± 0.21
*Actinobacteria*–*Bifidobacteriales*–*Bifidobacteriaceae* family						
*^a^ Bifidobacterium*	nd.	nd.	nd.	7.2 ± 0.03	nd.	nd.

Values represent mean ± S.D. Data for treatment groups are plotted against that for controls; * *p* ≤ 0.05; ** *p* ≤ 0.01; *** *p* ≤ 0.001. SD, Sandhoff disease; WT, wild-type. Notes: nd, not detected; *^a^* Mucolytic bacteria.

## Data Availability

The data presented in this study are available in manuscript.
